# Tailored Design of a Nanoporous Structure Suitable for Thick Si Electrodes on a Stiff Oxide-Based Solid Electrolyte

**DOI:** 10.1021/acsami.4c15894

**Published:** 2024-10-29

**Authors:** Kohei Marumoto, Kiyotaka Nakano, Yuki Kondo, Minoru Inaba, Takayuki Doi

**Affiliations:** † Department of Molecular Chemistry and Biochemistry, Doshisha University, Kyotanabe, Kyoto 610-0321, Japan; ‡ Development Department of CT Solution, Hitachi High-Tech Corporation, Hitachinaka, Ibaraki 312-0033, Japan

**Keywords:** pore size distribution, porosity, stress, garnet, lithium, all-solid-state battery

## Abstract

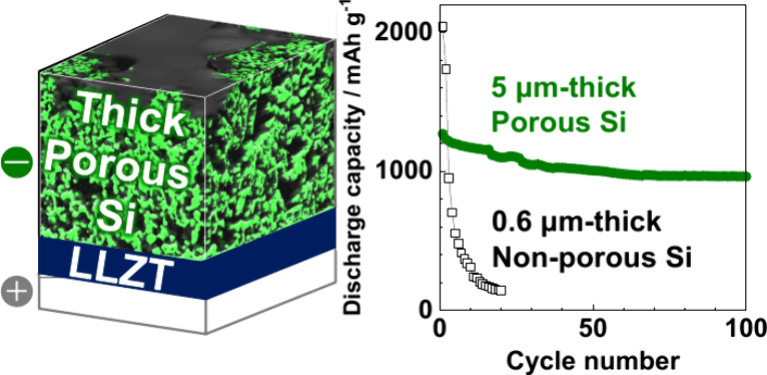

Oxide-based all-solid-state batteries are ideal next-generation batteries that combine high energy density and high safety, but their realization requires the development of interface bonding technology between the stiff solid electrolyte and electrode. Even if the interface could be bonded, it is difficult to hold the interface, because only the electrode expands/contracts unilaterally during charge/discharge reactions. In particular, silicon (Si), which has eagerly awaited as a next-generation negative-electrode material for many years, changes in volume by several hundred percent. To solve these problems, in this work, highly porous silicon oxide (SiO_*x*_) electrodes with different porous structures were fabricated on a stiff garnet-type Li_7_La_3_Zr_2_O_12_ solid electrolyte, the three-dimensional nanoporous structure was analyzed quantitatively, and the charge/discharge characteristics were investigated. The microscopic observation and electrochemical analysis revealed how we should control the porous structure, such as sizes of pores and SiO_*x*_, size distribution, and porosity, for repeated and stable charge/discharge cycles. In addition, the resultant porous SiO_*x*_ electrodes demonstrated superior charge/discharge cycle performance even when it thickened to 5 μm, whereas non-porous SiO_*x*_ easily peeled off from the solid electrolyte when its thickness exceeded 0.1 μm. The thick SiO_*x*_ films greatly improved the energy density per unit area (mAh cm^–2^). Nanosized fine pores with an interconnected open-pore architecture effectively mitigated the internal and interfacial stress upon expansion (charge)/contraction (discharge) of Si, and as a result, the thick and porous SiO_*x*_ electrode maintained the interfacial joint with the stiff solid electrolyte after repeated charge/discharge cycles. These results will provide useful insights for effectively designing more practical porous SiO_*x*_ powder effectively.

## Introduction

The particle sizes of active materials used in commercial lithium ion batteries (LIBs) typically range from a few micrometers to several tens of micrometers to achieve a high energy density. However, silicon (Si), which has attracted much attention as a next-generation negative-electrode material for LIBs, with such a large particle size can hardly repeat charge and discharge reactions.^1^ Si is alloyed/dealloyed with lithium (Li) in charge/discharge reactions, respectively, and the volume change reaches up to ca. 300%.^2,3^ The drastic expansion/contraction causes irreversible morphological changes, such as pulverization,^4^ and as a result, Si partly drops off to become inactive. Hence, the particle size of Si needs to be reduced to nanometer size to keep the absolute volume change small; e.g., a critical size of Si, where the cracking occurs through lithiation (charge)/delithiation (discharge) reactions with Li, is as small as 300 nm for the nanowires.^5^ When one-dimensionally nanosized Si, i.e., scale-shaped Si powder with 4 μm on a side, was used, the proper thickness was limited to ca. 100–200 nm.^6^ In liquid-type LIBs, even if the Si powder cracks, a new interface can be spontaneously formed as the electrolyte solution soaks into the cracks. However, this is not the case for all-solid-state batteries because the electrolyte is not fluid but a hard solid. The crack formation of Si can result in fatal collapse of the interfacial joint with a solid electrolyte. Accordingly, the internal stress of Si as well as the interfacial stress with a solid electrolyte must be reduced to prevent cracking.

All-solid-state LIBs can be broadly classified into two categories based on the type of solid electrolytes: oxide- and sulfide-based solid electrolytes. Because sulfide-based solid electrolytes are relatively plastic, they can easily form an interfacial joint with an electrode by applying a high pressure. Amorphous Si thin films, with a thickness of up to 300 nm, on a sulfide-based solid electrolyte exhibited high charge/discharge capacity and retained 85% of the initial capacity after 100 cycles.^7−10^ However, when the thickness increased further, the capacities rapidly decreased with repeated charge/discharge cycles due to the fatally irreversible morphological change of the Si electrode.^2,3^ The morphological stability can be improved by making Si porous.^11−13^ Sakabe et al. reported that a 4.72 μm thick porous Si film, having a closed pore structure and porosity of 40%, on a sulfide-based solid electrolyte achieved a high discharge capacity (2962 mAh g^–1^) and a high energy density (2.19 mAh cm^–2^) even after 100 charge/discharge cycles.^14^ The pores can effectively mitigate the interfacial stress between Si and the solid electrolyte as well as the internal stress of Si upon lithiation/delithiation reactions. However, the pore sizes were not specified, and there should be an optimal porous structure for the Si electrodes. Okuno et al. reported that porous Si powder also exhibited a high charge/discharge performance, while the loading mass was small at ca. 0.9 mg/cm^2^, and a very high pressure of 70–75 MPa was applied to the cell.^15^ The use of such a pressurization device reduces the energy density of batteries.

Oxide-based solid electrolytes, unlike sulfide-based electrolytes, have no risk of generating toxic gases, such as hydrogen sulfide, and hence, highly safe batteries should be achieved. Among the oxide-based solid electrolytes, garnet-type Li_7_La_3_Zr_2_O_12_ has particularly high stability against reduction, and thereby, Si and Li metal negative electrodes can be used.^16^ However, the oxide-based solid electrolytes are usually hard materials, like ceramics; the shear stress of oxide-based solid electrolytes (∼60 GPa) is much higher than that of sulfide-based electrolytes (∼10 Pa), making it far more difficult to form and keep an interfacial joint with an electrode.^17−19^ In fact, when the thickness of Si exceeded 100 nm, it easily peels off from the solid electrolyte, and the interface joint cannot be maintained after repeated charge/discharge cycles. Ping et al. reported that a 1 μm thick non-porous Si electrode on a garnet-type 3 wt % Al_2_O_3_-added Li_7_La_3_Zr_2_O_12_ (LLAZ) solid electrolyte exhibited a high discharge capacity of 2685 mAh g^–1^ and a high coulombic efficiency of 83.2% in the first cycle. The interface joint between Si and LLAZ was still maintained up to the second cycle, while the charge/discharge behavior after the third cycle was unknown.^20^ The successful charge–discharge of porous Si has not been reported thus far.

**Figure 1:**
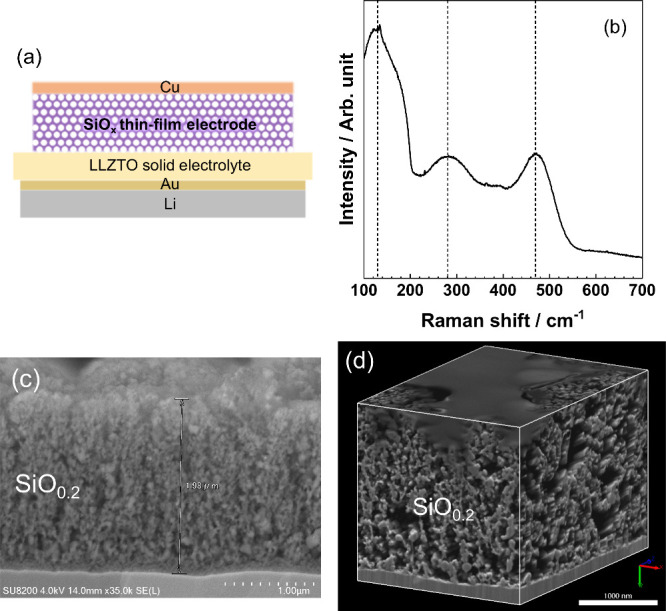
(a) Schematic of a SiO_*x*_|LLZTO|Li–Au cell, (b) Raman spectrum, (c) cross-sectional SEM image, and (d) FIB–SEM image of porous SiO_0.2_ films (sample 1).

Silicon oxide (SiO_*x*_) can deliver superior charge/discharge cycle performance to Si in liquid-type LIBs.^21−26^ SiO_*x*_ is known to have a composite structure consisting of a nanosized Si core with a SiO_*x*_ shell embedded in an amorphous SiO_2_ matrix.^27,28^ SiO_*x*_ and SiO_2_ reductively decompose to form lithium oxide (Li_2_O) and lithium silicate in the initial charge process.^29^ These decomposition products can act as buffers to mitigate the volume change of Si. Hence, SiO_*x*_ should be more suitable than Si even for all-solid-state LIBs. On the basis of these considerations, in this work, highly porous SiO_*x*_ thick films with different porous structures were deposited on a garnet-type Li_6.6_La_3_Zr_1.6_Ta_0.4_O_12_ (LLZTO) solid electrolyte by a radio-frequency (RF) sputtering method, and the correlation between the nanosized pore structure and charge/discharge properties was studied (Figure 1a).

## Results and Discussion

### Performance Limitation of Non-porous SiO_0.2_

Non-porous SiO_0.2_ thin-film electrodes with a thickness of about 100 nm are often used to study the charge/discharge characteristics of all-solid-state LIBs because it exhibited high charge/discharge capacities over repeated charge/discharge cycles, as shown in Figure S1 of the Supporting Information. However, the charge/discharge performance dramatically degraded with an increase in thickness; e.g., a 600 nm thick SiO_0.2_ electrode showed a rapid decrease in charge/discharge capacities within the first 10 cycles (Figure S1 of the Supporting Information) and peeled away from the LLZTO solid electrolyte. These results indicate that a unilateral volume change of Si upon lithiation and delithiation should cause an excessive interfacial stress between the SiO_0.2_ electrode and LLZTO electrolyte.

### Characterization of Porous SiO_0.2_

**Figure 2:**
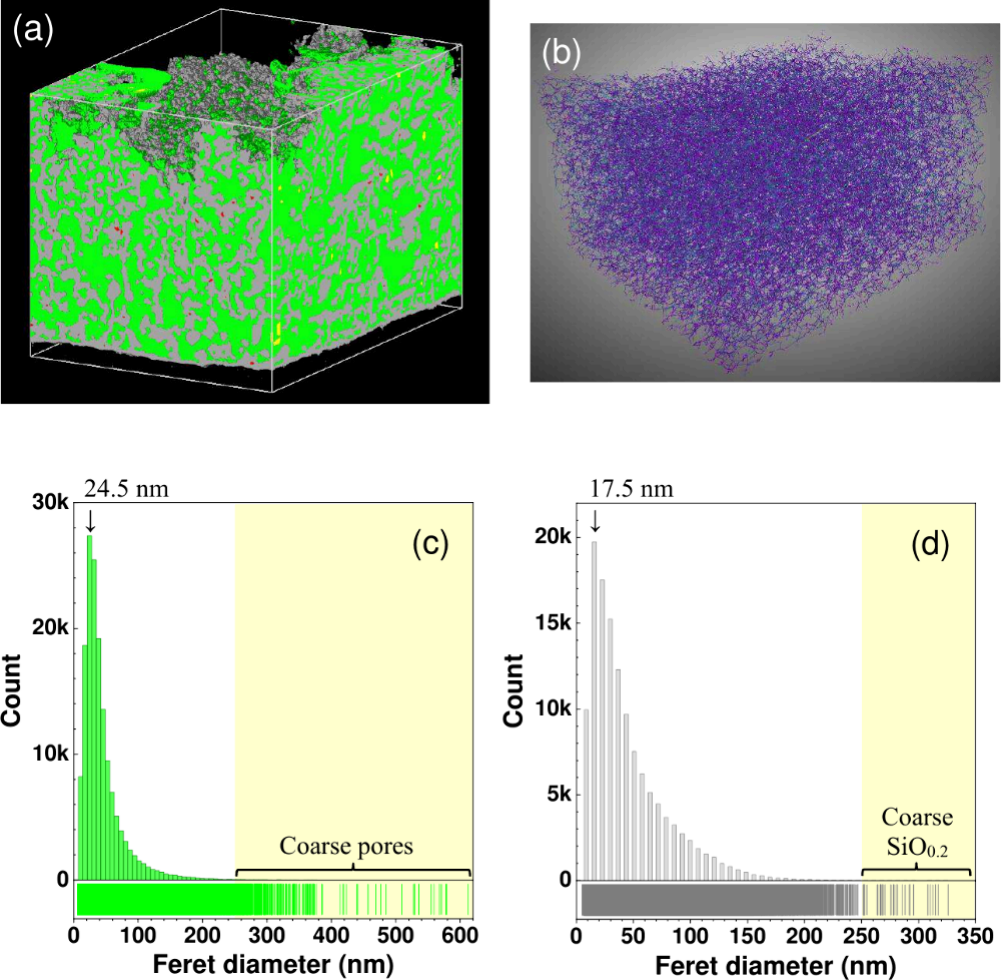
(a) Reassembled FIB–SEM image of a 2.0 μm thick porous SiO_0.2_ film in Figure 1d; gray, yellow, green, and red indicate interlinked SiO_0.2_, isolated SiO_0.2_, interlinked pore, and isolated pore, respectively. (b) Network of pores. Histograms of the mean Feret diameter of interlinked (c) pore and (d) SiO_0.2_.

To mitigate the internal and interfacial stress, porous SiO_0.2_ thin films were fabricated by RF magnetron sputtering in this work.^30^ The applied power was set to 200 and 50 W for Si and Zn, respectively. The resultant SiO_0.2_ thin film (sample 1) exhibited three broad peaks at around 140, 280, and 470 cm^–1^ and no band at 520 cm^–1^ for crystal Si in the Raman spectrum (Figure 1b), which suggests an amorphous structure.^31,32^ The cross-sectional image observed by field emission scanning electron microscopy (FE-SEM) clearly indicates that SiO_0.2_ was porous (Figure 1c). The composition was determined by FE-SEM/energy-dispersive X-ray spectroscopy (EDX). Si and O were uniformly present throughout the porous film, and little residual Zn was observed (panels a–d of Figure S2 of the Supporting Information); SiO_0.2_ was formed instead of Si because the vacuum chamber for thin-film fabrication was not airtight, and a small amount of air entered the chamber, causing silicon to be slightly oxidized. The more detailed morphology was observed by focused ion beam scanning electron microscopy (FIB–SEM; Figure 1d). A representative FIB–SEM image is shown in panels e and f of Figure S2 of the Supporting Information. The pores had an open structure with locally large pores, and SiO_0.2_ had a network structure with bottlenecks, which would prevent Li from diffusing easily. All of the resultant images were quantitatively analyzed using machine learning. Nanosized pores were observed throughout the SiO_0.2_ film (Figure 1d). The porosity reached as high as 53.6%, and 99.98% of the pores were connected with each other (panels a and b of Figure 2 and Table S1 of the Supporting Information). The pore sizes were widely distributed from 5.5 to 612 nm. Most of them were fine pores with Feret diameters of ca. 25 nm, while coarse pores larger than 250 nm were also present (Figure 2c). SiO_0.2_ was also largely interconnected (99.94%), and the remaining 0.06% would be connected with thin SiO_0.2_ below SEM resolution (5 nm/pixel). The sizes of SiO_0.2_ ranged from 5.5 to 326 nm. The Feret diameter peaked at ca. 18 nm, while coarse Si > 250 nm was also present (Figure 2d). Thus, most of the pores and SiO_0.2_ were approximately equal in size at around 20 nm, but coarse pores and SiO_0.2_ were also contained.

### Effect of the Nanosized Open-Pore Structure on Electrochemical Properties

**Figure 3:**
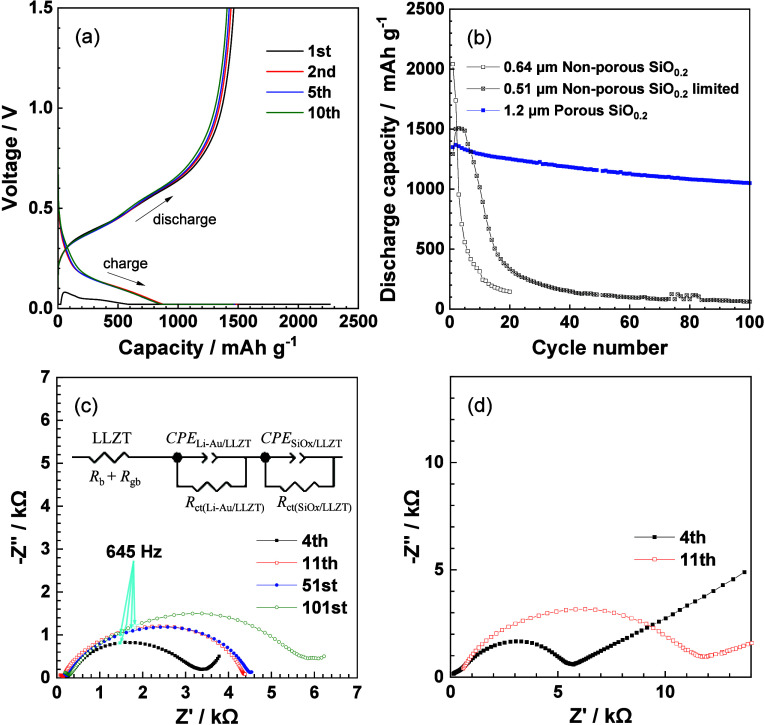
(a) Charge and discharge curves at C/10 rate and (c) Nyquist plots at 50% SOC of a Li–Au|LLZTO|1.2 μm thick porous SiO_0.2_ film (7 mm ⌀) cell. (b) Variation of discharge capacities at C/10 rate with the cycle number of Li–Au|LLZTO|SiO_0.2_ cells using 0.64 and 0.51 μm thick non-porous SiO_0.2_ films and a 1.2 μm thick porous SiO_0.2_ film. The discharge capacity of the 0.51 μm thick non-porous SiO_0.2_ film was limited to 1500 mAh g^–1^. (d) Nyquist plots at 50% SOC of a Li–Au|LLZTO|non-porous 0.64 μm thick SiO_0.2_ (7 mm ⌀) cell.

The thin-film electrodes used were not subject to any pressing process, and hence, the SEM images in Figures 1 and 2 represent the actual pore structure of the porous SiO_0.2_ electrode that was incorporated in a cell. The initial charge reactions of the 1.2 μm thick porous SiO_0.2_ electrode (sample 1) proceeded at a voltage below 0.1 V (Figure 3a), and the polarization was larger than that in the second and subsequent cycles. The resultant porous film was not pure silicon but silicon oxide, and therefore, the irreversible decomposition reaction should occur during the first charge. In addition, the long pathways for electronic conduction and lithium diffusion due to the porous structure would also be a factor in the large polarization. In the subsequent initial discharge process, a typical discharge curve of an amorphous silicon electrode was observed, and it delivered a high discharge capacity of 1349 mAh g^–1^ at a C/10 rate (Figure 3a) and retained 77.8% of it even after 100 cycles (Figure 3b). However, the initial coulombic efficiency was low compared to non-porous SiO_0.2_ electrodes, and the average coulombic efficiency in 100 cycles remained at 92.5% (Figure S3 of the Supporting Information). The reason for these is not understood. A 0.64 μm thick non-porous SiO_0.2_ electrode (sample 2), having an equal loading with the 1.2 μm thick sample 1 (0.13 mg/cm^2^), exhibited the higher discharge capacity in the initial cycle (2041 mAh g^–1^), while it rapidly decreased within the 10 cycles. The capacity fading could not be improved even when the discharge capacity was controlled to the same level (1500 mAh g^–1^) as the 1.2 μm thick sample 1 (Figure 3b). Thus, the charge–discharge performance was greatly improved by making SiO_0.2_ porous. On the other hand, when electrode materials are made porous, the volume increases due to the presence of pores, and hence, the volumetric capacity in the initial cycle became low (Table S2 of the Supporting Information). However, the non-porous electrode showed a severe capacity drop, and hence, the porous electrode showed a rather higher volumetric capacity after repeated charge/discharge cycles.

**Figure 4:**
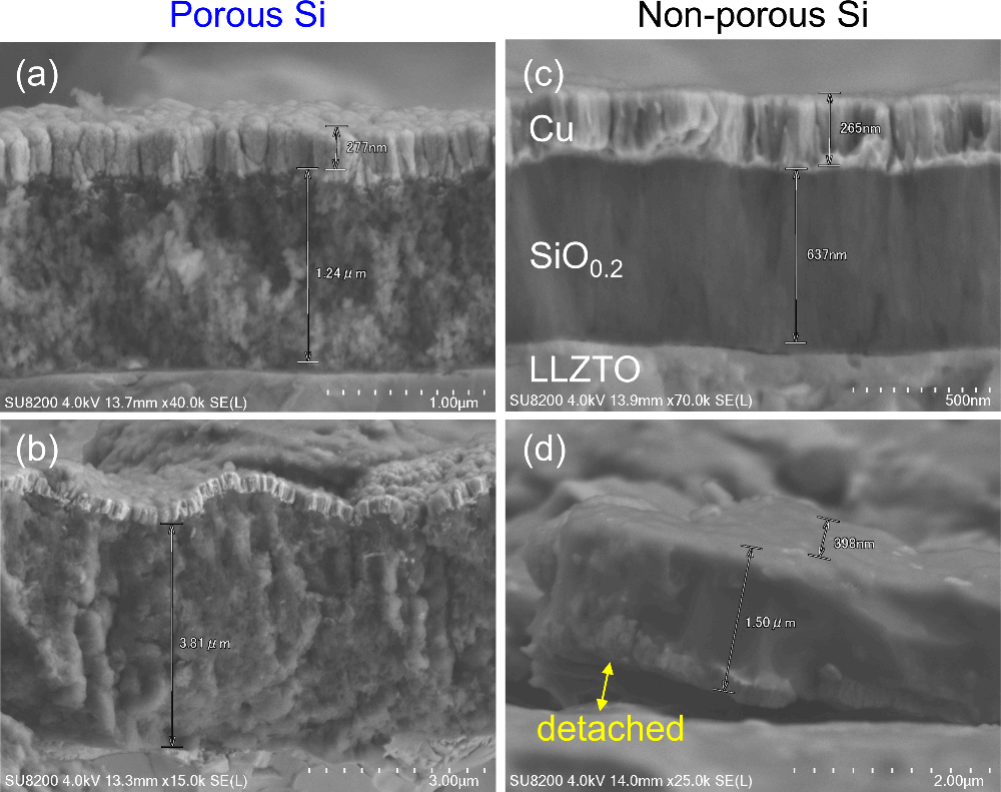
Cross-sectional SEM images of (a and b) Li–Au|LLZTO|1.2 μm thick porous SiO_0.2_ and (c and d) Li–Au|LLZTO|0.64 μm thick non-porous SiO_0.2_ cells (a and c) before and after (b) 100 and (d) 20 cycles.

To gain a deeper understanding of the effect of the nanosized pores on the charge/discharge reactions, the internal resistance of cells was investigated by alternating current (AC) impedance spectroscopy. In the fourth cycle, porous SiO_0.2_ (sample 1; Figure 3c) gave lower interfacial resistance than non-porous SiO_0.2_ (sample 2; Figure 3d). The former remained low even at the 101th cycle, whereas the latter significantly increased at the 11th cycle (Table S3 of the Supporting Information).^33^ Thus, it is relatively easier for Li^+^ to transfer at an interface between porous SiO_0.2_ and LLZTO, and therefore, high discharge capacities were retained in 100 cycles (Figure 3b). The cells were cut after repeated charge–discharge cycles to observe the morphological changes of the cross sections. Porous SiO_0.2_ (sample 1) well maintained the interfacial joint after 100 cycles, whereas the thickness increased by 3.2 times from 1.2 to 3.8 μm (panels a and b of Figure 4). A more careful observation revealed that the porous structure was still seen after 100 cycles, but the microstructure was quite different from the original structure; fine particles with a diameter of ca. 200 nm formed the porous structure, and several microcracks appeared in a vertical direction of the electrode (Figure 4b), as is often seen in the literature.^34^ These results can be explained as follows: during charging, the porous SiO_0.2_ electrode expands due to alloying with Li and the pores disappear. In the subsequent discharge process, Li diffuses vertically to the electrode to dealloy and the contraction stress caused vertical cracking. The resultant critical grain size was found to be about 200 nm. More detailed morphological changes should be clarified by *in situ* SEM observation. On the other hand, non-porous SiO_0.2_ (sample 2) partly exfoliated from LLZTO after the 20th cycle (panels c and d of Figure 4), which was consistent with a drastic decline in discharge capacity (Figure 3b) and an abrupt rise in internal resistance (Figure 3d). Thus, the original porous structure collapsed through the repeated expansion/contraction of SiO_0.2_, while the pores remained to serve as a buffer against the internal and interfacial stresses and to maintain the interfacial joint.

### Thicker and Porous SiO_0.2_ Electrodes

**Figure 5:**
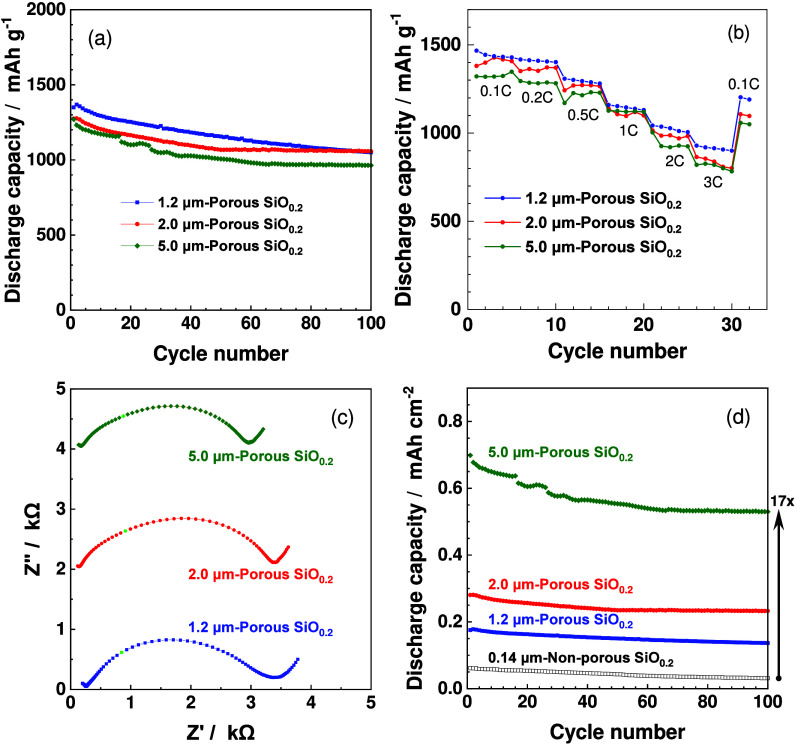
Variation of gravimetric discharge capacities (mAh g^–1^) with (a) cycle number and (b) discharge rates of Li–Au|LLZTO|porous SiO_0.2_ film cells. (c) Nyquist plots of Li–Au|LLZTO|porous SiO_0.2_ (7 mm ⌀) cells at 50% SOC in the fourth cycle. (d) Variation of areal discharge capacities (mAh cm^–2^) with cycle number of Li–Au|LLZTO|porous SiO_0.2_ film cells.

The thickness of porous SiO_0.2_ (sample 1) was increased further up to 5.0 μm (Figure 5a). The 2.0 and 5.0 μm thick SiO_0.2_ electrodes also achieved high discharge capacity retention after 100 cycles, 82.9 and 75.9%, respectively. The thick SiO_0.2_ electrodes delivered high discharge capacities of more than 800 mAh g^–1^ even at a high discharge rate of 3 C, which corresponds to a current that can theoretically complete each charge (discharge) process in ^1^/_3_ h (20 min) under the presumption that the theoretical specific capacity of SiO_0.2_ is 4199 mAh/g (Figure 5b). The high rate performance was maintained even with thicker SiO_0.2_ films, which was supported by the fact that the internal resistance of the cells was nearly identical regardless of SiO_0.2_ film thickness (Figure 5c). Although the pathways for electronic conduction and lithium diffusion lengthened with increasing the film thickness, the interfacial resistance and rate characteristics did not decrease. Therefore, the interfacial lithium ion transfer process between the electrode and electrolyte is considered to be the rate-limiting step in discharge reactions. The interfacial joint was well-maintained even for the thick Si electrodes to achieve the high charge/discharge cycle performance. Accordingly, the discharge capacity per unit area (mAh cm^–2^) remarkably increased with an increase in the thickness of porous SiO_0.2_ (Figure 5d). Thus, the energy density of SiO_0.2_ electrodes increased by making it porous and thick. It will be possible to further thicken porous SiO_0.2_ beyond 5 μm.

### Design of a More Suitable Pore Structure

**Figure 6:**
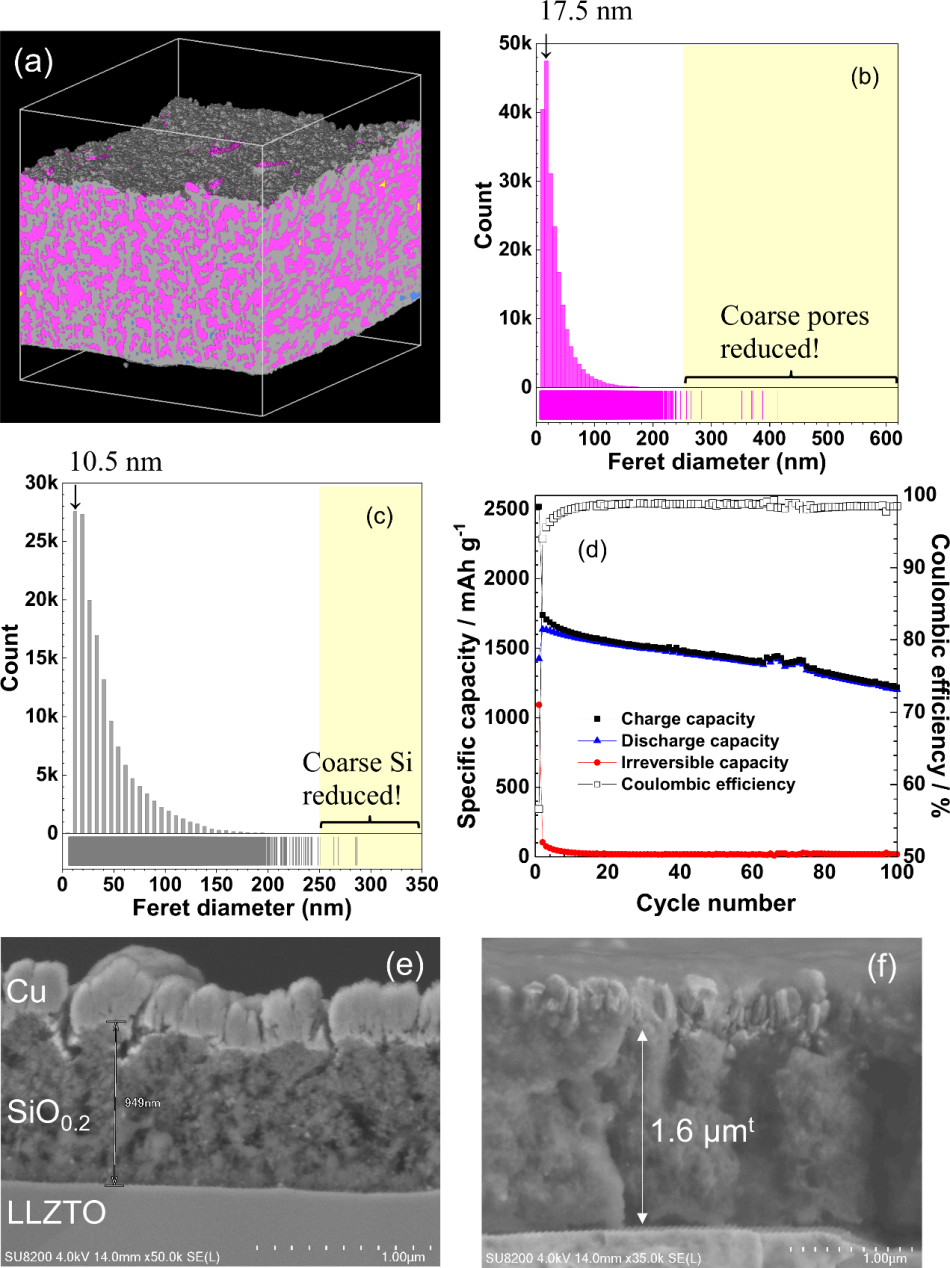
(a) Reassembled FIB–SEM image of a 1.2 μm thick porous SiO_0.2_ film (sample 3); gray, yellow, pink, and blue indicate interlinked SiO_0.2_, isolated SiO_0.2_, interlinked pore, and isolated pore, respectively. Histograms of the mean Feret diameter of interlinked (b) pore and (c) SiO_0.2_. (d) Variation of charge/discharge capacities and coulombic efficiencies of a 0.95 μm thick porous SiO_0.2_ film (sample 3). Cross-sectional SEM images of Li–Au|LLZTO|0.95 μm thick porous SiO_0.2_ cells (e) before and (f) after 100 cycles.

Porous SiO_0.2_ with a finer pore structure was fabricated by RF sputtering (sample 3; Figure 6a); the applied power was decreased to 150 and 25 W for Si and Zn, respectively. The coarse pores with sizes larger than 250 nm were reduced, and the modal diameter was lowered to ca. 18 nm (Figure 6b) compared to sample 1 (Figure 2c). Most of the pores in sample 3, like sample 1, were connected with each other to form a three-dimensional open pore network. Coarse SiO_0.2_ was also relatively reduced, and the modal diameter decreased to ca. 11 nm (Figure 6c). However, the porosity of sample 3 (42.6%) was lower than sample 1 (53.6%). The charge/discharge performance of SiO_0.2_ is greatly affected by the loading mass (mg/cm^2^), as noted in Figure S1 of the Supporting Information. To compare the charge/discharge characteristics fairly with sample 1, porous SiO_0.2_ with almost the same loading mass was used; i.e., the loading mass of SiO_0.2_ in samples 1 and 3 was 0.13 and 0.12 mg/cm^2^, respectively. Because the porosity was different from each other, the thicknesses of samples 1 and 3 were 1.2 and 0.95 μm, respectively. The 0.95 μm thick sample 3 exhibited higher discharge capacities than sample 1, as shown in Figure 6d, and the average coulombic efficiency improved to 98.0%. These results clearly indicate that more SiO_0.2_ can be involved in charge and discharge reactions by making the porous SiO_0.2_ structure fine and that the generation of inactive SiO_0.2_ can be suppressed. The effect of the structural refinement was more evident in the morphological changes of electrodes; the increase in thickness of sample 3 after 100 cycles was reduced to 1.7 times (from 0.95 to 1.6 μm), as shown in panels e and f of Figure 6, despite the lower porosity than that of sample 1. These results suggest that forming more uniform and finer pores should be more effective for stable charge–discharge cycling of porous SiO_0.2_ than increasing the porosity. Thus, porous SiO_0.2_ with as many uniform and fine pores as possible, whether in thin films or powder, is considered suitable for all-solid-state batteries. The synthesis method of porous SiO_0.2_ films in this study can be applied to powder synthesis, which allows for the fabrication of practical composite electrodes using porous SiO_0.2_ powder. Thus, the present results can lead to a practical technology for realizing high-energy-density oxide-based all-solid-state batteries.

## Conclusion

A SiO_0.2_ negative electrode on a stiff LLZTO solid electrolyte can be stably charged and discharged by making it porous. The porous structure, such as sizes of pores and SiO_0.2_, their distribution, and porosity, was quantitatively analyzed by FIB–SEM to determine the effect of them on the charge/discharge reactions of SiO_0.2_. Resultant porous SiO_0.2_ had interconnected and open pores. The microscopic observation and electrochemical analysis revealed that the reduction of coarse pores and SiO_0.2_ larger than 250 nm allowed porous SiO_0.2_ to be charged/discharged efficiently. Reducing the pore size and narrowing its distribution were more effective in improving the charge/discharge cycle performance than increasing the porosity. There is room for further research to control the pore structure by optimizing the sputtering conditions, such as applied voltage, substrate temperature, gas atmosphere, and its pressure. The stable charge/discharge cycling was achieved even when the thickness of porous SiO_0.2_ was increased to 5.0 μm, whereas non-porous SiO_0.2_ thicker than 0.1 μm easily peeled off from the LLZTO solid electrolyte within 10 charge/discharge cycles. The porous SiO_0.2_ electrode maintained the interfacial joint with LLZTO, and the interfacial resistance between SiO_0.2_ and LLZTO was kept low. The pores remained after repeated charge/discharge cycles. These results indicate that nanosized pores with interconnected open-pore architecture effectively served as a buffer to mitigate the internal and interfacial stress upon expansion (lithiation)/contraction (delithiation) of Si, and as a result, the interfacial Li^+^ transfer reactions could proceed over repeated charge/discharge cycles. The discharge capacity per unit area (mAh cm^–2^) increased with an increase in thickness of porous SiO_0.2_, and 5.0 μm thick SiO_0.2_ exhibited approximately 17 times higher energy density than non-porous SiO_0.2_ with a typical thickness of ca. 0.1 μm. It will be possible to thicken porous SiO_0.2_ beyond 5 μm and improve the charge/discharge performance and energy density by tailoring the nanoporous structure further. These findings will make a new departure to the design of Si powder for realizing high-energy-density oxide-based all-solid-state batteries.

## Experimental Section

### Preparation of Porous SiO_0.2_ Thick Films

Porous SiO_0.2_ films were prepared on a LLZTO disk or Cu foil by a RF sputtering method. A 0.5 mm thick LLZTO disk was fabricated by a typical solid-state sintering method, as previously reported.^35^ The LLZTO disk (15 mm ⌀) was masked with polyimide tape to expose an area of 7 mm ⌀. The chamber was evacuated to less than 3.0 × 10^–4^ Pa, and the temperature of a LLZTO substrate was set to 200 °C. Then, boron-doped Si (>99.999%, 100 mm ⌀, 5 mm thickness, Toyoshima MFG) and single-crystal Zn (>99.99%, 75 mm ⌀, 5 mm thickness, Toyoshima MFG) targets were co-sputtered at 1.0 Pa under 30 standard cubic centimeters per minute (sccm) flow of an Ar gas. The applied power was set to 150–200 and 25–50 W for Si and Zn, respectively. The thickness of the resultant Si–Zn film was adjusted by controlling the deposition time. After the deposition, the Ar gas flow was stopped and the chamber was evacuated to less than 3.0 × 10^–4^ Pa. The substrate temperature was raised to 550 °C to remove Zn. A Cu thin film was deposited on the porous SiO_0.2_ film as a current collector using a Cu target (>99.99%, 75 mm ⌀, 5 mm thickness, Toyoshima MFG). Non-porous SiO_0.2_ thin films were also prepared by sputtering the boron-doped Si target for reference.

### Characterization of Porous SiO_0.2_ Thick Films

The crystal structure of a porous SiO_0.2_ thin film was analyzed by Raman spectroscopy (LabRAM HR Evolution, Horiba). Raman spectra were obtained at room temperature by excitation with a 785 nm laser (10 mW). The integration time was set to 300 s × 5 times. The resultant LLZTO disk with a porous Si thin film was broken by hand to observe the cross sections by FE-SEM (SU8220, Hitachi High-Tech) equipped with EDX (XFlash6|10, Bruker). The absence of residual Zn in the porous SiO_0.2_ films was confirmed by SEM/EDX analysis. A more detailed porous structure was investigated by FIB–SEM (NX9000, Hitachi High-Tech). A field of view with a size of 2.5 μm square was etched by FIB at 5 nm intervals in a *Z* direction (Figure 2a) to observe the cross sections by SEM. This “cut and see” process was conducted repeatedly to acquire 651 cross-sectional images. The resultant SEM images were constructed to obtain a three-dimensional (3D) image. In addition, the moiety of SiO_0.2_ and pore in seven cross-sectional SEM images was identified manually to obtain teaching data, and then the whole image was segmented by machine learning using Dragonfly (Comet Technologies Canada, Inc.) and manual fine adjustment; Dragonfly used artificial intelligence (AI) functions to perform the segmentation based on the above teaching data. The 3D reconstruction from two-dimensional (2D) images was conducted by Dragonfly. The Feret diameters of SiO_0.2_ and pore were evaluated from the sliced images. The connectivity of the pores was confirmed by connecting the interlinked pores in a network.

### Electrochemical Measurements of Porous SiO_0.2_ Electrodes

To improve the wettability of the Li metal counter electrode and LLZTO solid electrolyte and suppress the reaction distribution in the plane, a thin gold layer was placed between them. A 30 nm thick Au film (13 mm ⌀) was deposited on the other side of the LLZTO disk by direct current (DC) magnetron sputtering (MSP-10, Vacuum device). It was transferred to an Ar-filled glovebox with a dew point below −70 °C (Miwa MFG). A 0.5 mm thick Li foil (10 mm ⌀, Honjo Metal) was attached to the Au film and then placed in a cell body that is typically used for charge–discharge tests of liquid-type LIBs. Hence, the Li–Au|LLZTO|SiO_0.2_ laminate was held under a moderate pressure of 1180 kPa in a vertical direction; i.e., the thin-film electrodes were tested without any pressing process. The charge and discharge tests were performed at 30 °C with a charge–discharge system (TOSCAT-3100, Toyo System) between 1.50 and 0.02 V at a C/10 rate, which corresponds to a current that can theoretically complete each charge (discharge) process of the theoretical capacity (4200 mAh g^–1^) in 10 h.^1^ In each charge process, after the charging voltage reached 0.02 V, the cell was subsequently held at this voltage until the current decayed to 0.01 C. At the end of each charge/discharge process, the cell was rested under an open circuit state for 10 min. AC impedance spectroscopy (SP-200 and VSP300, BioLogic) was performed at 50% state of charge (SOC) in given cycles by applying a sine wave with an amplitude of 10 mV over the frequency range from 7 MHz to 0.5 Hz at 30 °C. The interfacial resistance at Li–Au|LLZTO was evaluated by measuring a Li–Au|LLZTO|Li–Au symmetrical cell after given charge–discharge cycles (Figure S4 of the Supporting Information). These preliminarily determined values were used to evaluate the interfacial resistance at SiO_0.2_|LLZTO from the Nyquist plots of Li–Au|LLZTO|SiO_0.2_ cells. In the rate performance test, the discharge rate was changed every 5 cycles from 0.1 to 3 C while performing each charge process at a constant rate of 0.1 C.

## Supporting Information Available

Volume fraction of the pore and SiO_0.2_, interfacial resistivity at SiO_0.2_ and LLZTO, charge/discharge cycle performance of non-porous SiO_0.2_, coulombic efficiency of SiO_0.2_, and Nyquist plots of a Li–Au|LLZTO|Li–Au symmetrical cell (PDF)
