# Supplementary material for: Tailored Design of a Nanoporous Structure Suitable for Thick Si Electrodes on a Stiff Oxide-Based Solid Electrolyte
Source: ACS Appl Mater Interfaces. 2024 Oct 29;16(45):62274–81. doi: 10.1021/acsami.4c15894 (PMC11586901; doi:10.1021/acsami.4c15894)
Supplement: Supplementary file 1 — am4c15894_si_001.pdf [file am4c15894_si_001.pdf]

Supporting information

Tailored Design of Nanoporous Structure Suitable for Thick Si Electrodes on Stiff Oxide-based Solid Electrolyte

*Kohei Marumoto [a], Kiyotaka Nakano [b], Yuki Kondo [a], Minoru Inaba [a], and Takayuki Doi [a]**

[a] *Department of Molecular Chemistry and Biochemistry, Doshisha University, Kyotanabe, Kyoto 610-0321, Japan*

[b] *Development department of CT Solution, Hitachi High-Tech Corporation, Hitachinaka, Ibaraki 312-0033, Japan.*

Email: tdoi@mail.doshisha.ac.jp

Table S1 Occupied volume of Si and pore in a 2.0 μm-thick porous $SiO_{0.2}$ film (Sample 1)

		Volume (nm^3)	Fraction (%)	
$SiO_{0.2}$	Interconnected $SiO_{0.2}$	7,014,476,746.25	99.94%	46.4%
	Isolated $SiO_{0.2}$	4,561,593.75	0.06%	
Pore	Interlinked pore	8,105,685,343.75	99.98%	53.6%
	Isolated pore	1,259,906.25	0.02%	

Table S2 Volumetric discharge capacity (mAh cm^{-3}) of Li-Au | LLZTO | $SiO_{0.2}$ cells using 0.64 μm-thick non-porous- and 1.2 μm-thick porous-$SiO_{0.2}$ films. The diameter of the $SiO_{0.2}$ electrodes was 7 mm.

$SiO_{0.2}$	1st cycle			After charge/discharge cycles		
	Initial thickness (μm)*	Discharge capacity (mAh g^{-1})**	Discharge capacity (mAh cm^{-3})	Thickness (μm)*	Discharge capacity (mAh g^{-1})**	Discharge capacity (mAh cm^{-3})
Non-porous	0.64	1292	3063	20th cycle		
				1.5	145	147
Porous	1.24	1349	1484	100th cycle		
				3.8	1050	365

* Electrode thickeness in Figure **4**. ** Gravimetric discharge capacity in Figure **3b**.

Table S3 Interfacial resistivity (Ω cm^2) at 1.2 μm-thick porous $SiO_{0.2}$ film (7 mmϕ) | LLZTO and 0.64 μm-thick non-porous $SiO_{0.2}$ (7 mmϕ) | LLZTO evaluated from Figures **3c, 3d** and **S4**.

Interfacial resistivity (Ω cm^2)	4th	11th	51st	101st
1.2 μm porous Si | LLZTO (Figure **3c**)	1163	1670	1500	2266
0.64 μm non-porous Si | LLZTO (Figure **3d**)	2298	4437	-	-

The interfacial resistivity decreased by making $SiO_{0.2}$ porous, while it was still higher than that for a $SiO_{0.2}$ | electrolyte solution interface; e.g., 58.9 Ω cm^2 when 1 M $LiPF_6$ dissolved in a mixture of ethylene carbonate and diethyl carbonate (1:1 by vol.) was used as an electrolyte solution.

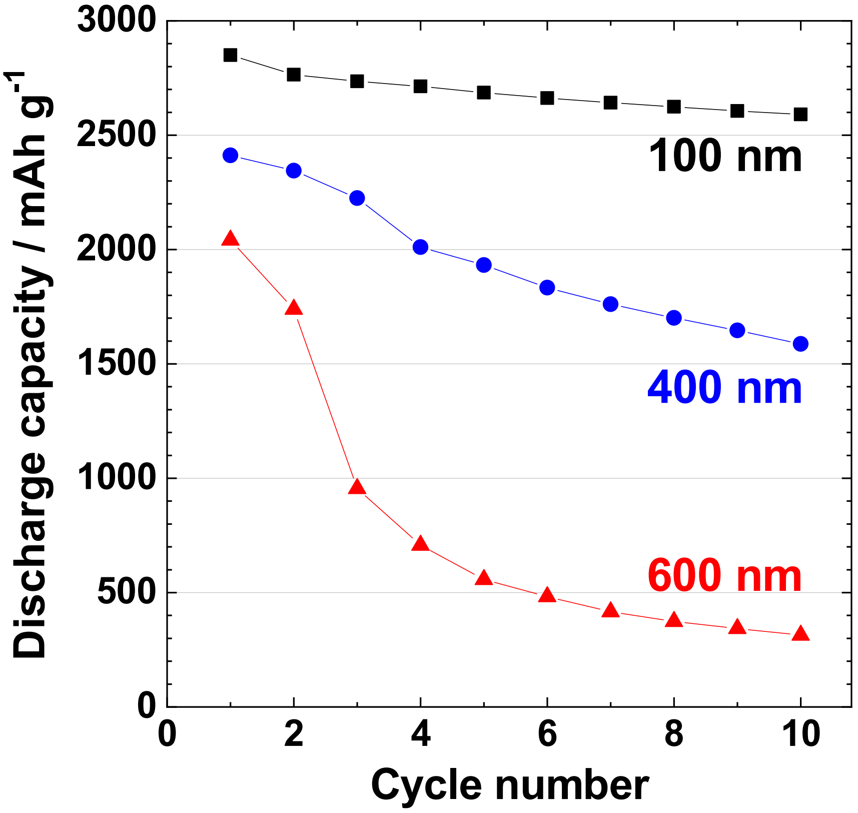

Figure S1. Variation of discharge capacities with cycle number of Li-Au | LLZTO | $SiO_{0.2}$ cells using 100, 400 and 600 nm-thick non-porous $SiO_{0.2}$ films that were deposited by a radio frequency (RF) sputtering method.

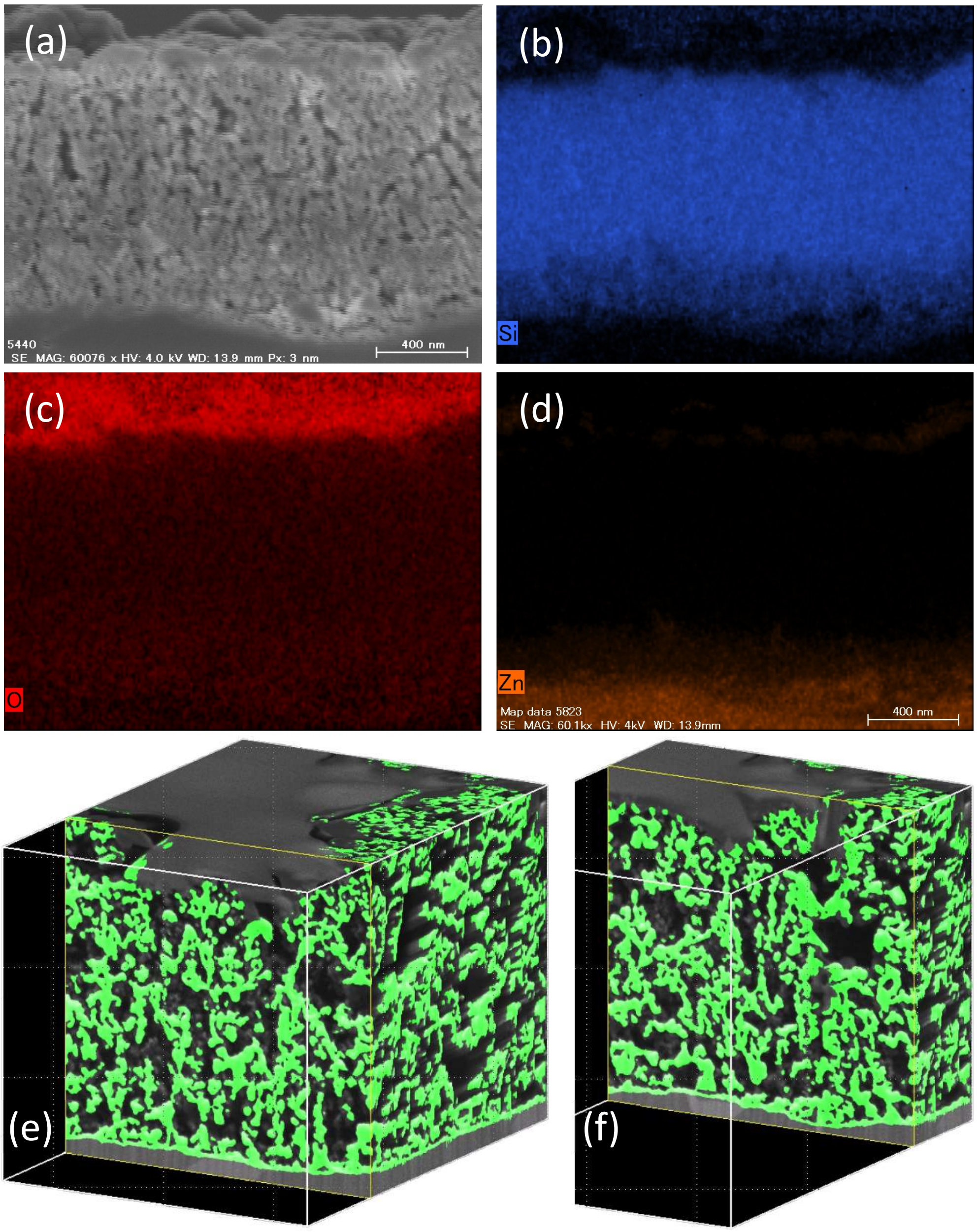

Figure S2. Cross-sectional (a) SEM, (b-d) SEM/EDX, (e and f) FIB-SEM images of porous $SiO_{0.2}$ films (Sample 1).

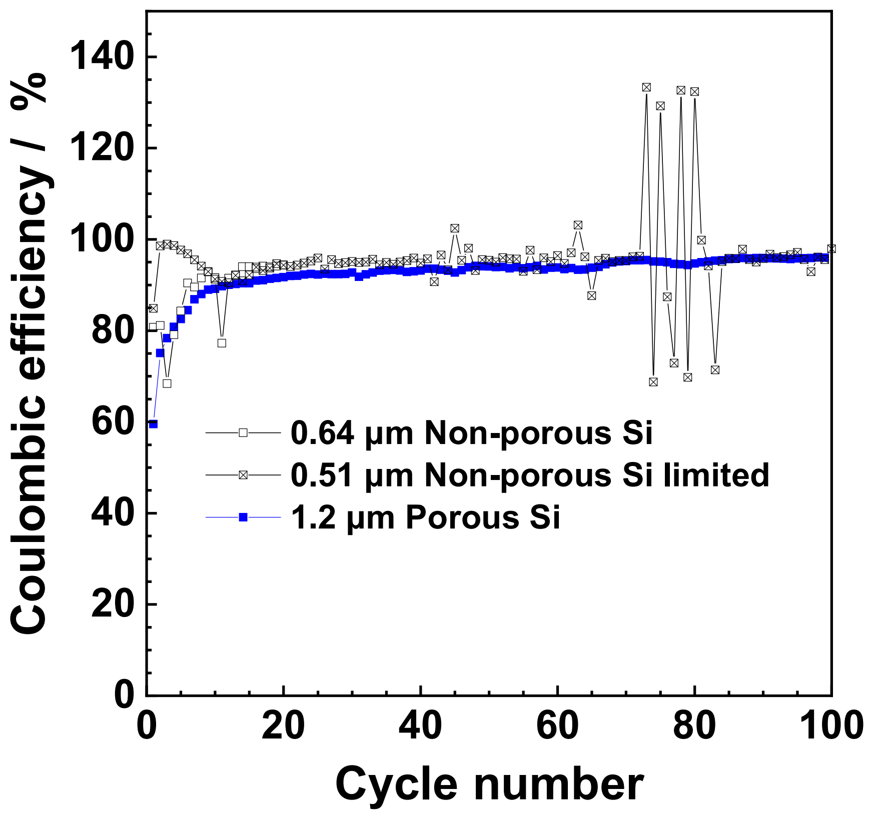

Figure S3. Variation of Coulombic efficiencies with cycle number of Li-Au | LLZTO | $SiO_{0.2}$ cells using 0.64 μm- and 0.51 μm-thick non-porous $SiO_{0.2}$ films, and a 1.2 μm-thick porous $SiO_{0.2}$ film.

The average Coulombic efficiencies of 0.64 μm- and 0.51 μm-thick non-porous $SiO_{0.2}$ films, and a 1.2 μm-thick porous $SiO_{0.2}$ film were 88.4 % in 20 cycles, 95.6% in 100 cycles, and 92.5% in 100 cycles, respectively.

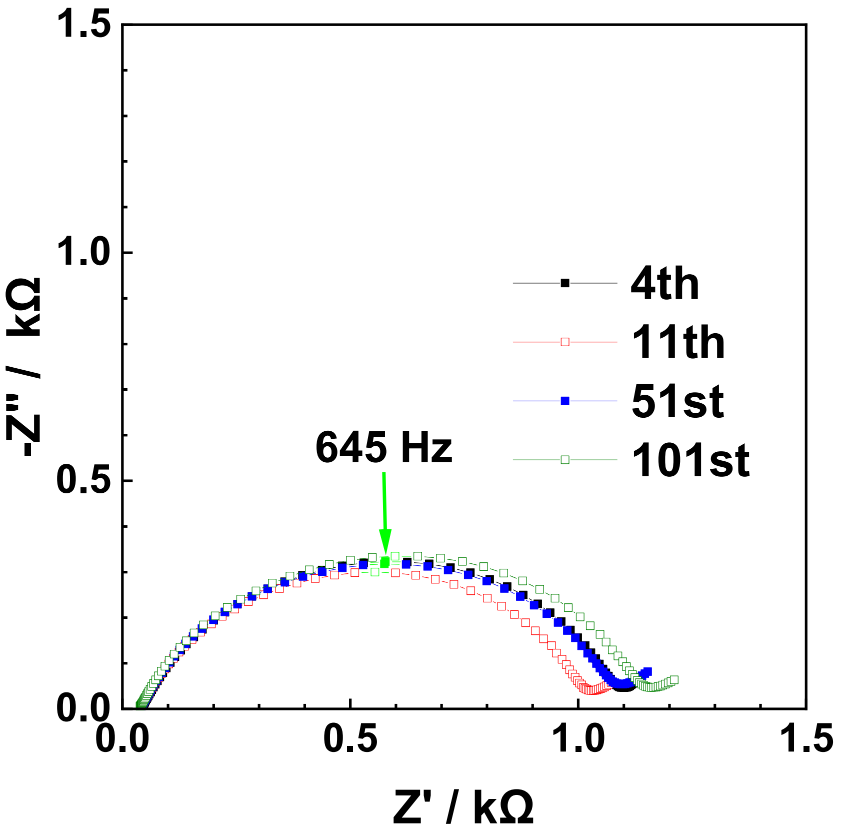

Figure S4. Nyquist plots of a Li-Au | LLZTO | Li-Au symmetrical cell after the 4th, 11th, 51st, and 101st charge/discharge cycles. The charge/discharge tests were conducted at the same current density (47.02 μA cm^{-2}) and the almost same capacity (1st-10th: 0.10676 mAh, 11th-50th: 0.0885 mAh, 51st-100th: 0.0865 mAh) as a porous 2.0 μm-thick $SiO_{0.2}$ | LLZTO | Li-Au cell.

The characteristic frequency was ca. 645 Hz. The interfacial resistance increased slightly with cycling, but not significantly. Based on these preliminarily results, the $SiO_{0.2}$ | LLZTO interfacial resistance was evaluated from Figures **3c** and **3d**.
